# Public awareness, knowledge of availability, and willingness to use neurosurgical care services in Sub-Saharan Africa: A cross-sectional study

**DOI:** 10.1371/journal.pone.0264955

**Published:** 2022-03-17

**Authors:** Chibuikem A. Ikwuegbuenyi, Alice Umutoni, Neri Ngole Atabe Ngwene, Placide Ngoma, Arsene Daniel Nyalundja, Daniel Safari Nteranya, Tunde A. Olobatoke, Oloruntoba Ogunfolaji, Dawin Sichimba, Joanitor Najjuma, Lorraine Arabang Sebopelo, Aliyu Ndajiwo, Michael A. Bamimore, Gideon Adegboyega, Ulrick Sidney Kanmounye

**Affiliations:** Research Department, Association of Future African Neurosurgeons, Yaounde, Cameroon; Mayo Clinic, UNITED STATES

## Abstract

**Introduction:**

Low- and middle-income countries bear the majority of neurosurgical disease burden and patients face significant barriers to seeking, reaching, and receiving care. We aimed to understand barriers to seeking care among adult Africans by evaluating the public perception, knowledge of availability, and readiness to use neurosurgical care services.

**Methods:**

An e-survey was distributed among African adults who are not in the health sector or pursuing a health-related degree. Chi-square test and ANOVA were used for bivariate analysis and the alpha value was set at 0.05. Odds ratios and their 95% confidence intervals were calculated.

**Results:**

Six hundred and sixty-two adults from 16 African countries aged 25.4 (95% CI: 25.0, 25.9) responded. The majority lived in urban settings (90.6%) and were English-speaking (76.4%) men (54.8%). Most respondents (76.3%) could define neurosurgery adequately. The most popular neurosurgical diseases were traumatic brain injury (76.3%), congenital brain and spine diseases (67.7%), and stroke (60.4%). Unwillingness to use or recommend in-country neurosurgical services was associated with rural dwelling (β = -0.69, SE = 0.31, P = 0.03), lack of awareness about the availability of neurosurgeons in-country (β = 1.02, SE = 0.20, P<0.001), and believing neurosurgery is expensive (β = -1.49, SE = 0.36, P<0.001).

**Conclusion:**

Knowledge levels about neurosurgery are satisfactory; however, healthcare-seeking is negatively impacted by multiple factors.

## Introduction

Over 18 million people die every year from the absence of essential and emergency surgical care, the majority of these deaths have been shown to occur in low- and middle-income countries (LMICs) [1]. This is especially true in Africa where the disease burden is compounded by a lack of resources and numerous barriers to care [2]. In recent years, there has been increased attention on the global health needs of Africa; however, the focus has been on infectious and non-communicable diseases [3]. As a result, resource allocation for surgical specialties like neurosurgery is suboptimal [1], despite surgical and neurological disorders being a leading cause of death and disability [3]. This discrepancy is best illustrated by the unmet neurosurgical need. Africa accounts for 15% of the unmet global neurosurgical need but African patients have access to only 1% of the neurosurgical workforce [3].

Large-scale, comprehensive, and coordinated public health efforts are needed to reduce the burden of neurosurgical diseases and barriers to care [4]. Barriers to care can be classified according to the health system components they affect or based on the patient continuum of care (i.e., barriers to seeking, reaching, and receiving care). The most affected health system components are infrastructure and workforce [5]. For example, African neurosurgeons report limited access to essential equipment such as computed tomography (CT), magnetic resonance imaging (MRI), operative microscopes, and surgical consumables [5]. In addition, Africa has the lowest neurosurgical workforce density worldwide with around 1 neurosurgeon per 2 000 000 population [1]. This density is well below the recommended target of 1 specialist neurosurgeon per 200 000 people [1]. African patients face other barriers to care such as the concentration of neurosurgical infrastructures and personnel in urban areas, lack of financial risk protection, and lack of awareness [6].

Healthcare-seeking behavior (HSB) is an important determinant of barriers to seeking care. HSB is defined as “any action or inaction undertaken by individuals who perceive themselves to have a health problem or to be ill to find an appropriate remedy” [7]. HSB is determined by a person’s characteristics and behaviors, physical environment, socio-economic environment, health cost, and availability [8, 9]. For example, Ethiopian and Nigerian patients believe cranial diseases are supernatural and resort to traditional medicine or spiritual healing before consulting neurosurgeons [10]. Hence, inappropriate HSBs and misconceptions of neurosurgery form considerable barriers to seeking care. With that in mind, we aimed to understand the public awareness, knowledge of availability, and readiness of neurosurgical care services among adult laypersons in Africa.

## Materials and methods

This e-survey was reported in accordance with the Checklist for Reporting Results of Internet E-Surveys (CHERRIES), STrengthening the Reporting Of Cohort Studies in Surgery (STROCSS), and Strengthening the Reporting of Observational Studies in Epidemiology (STROBE) [11–13].

### Study design

The study methodology followed a pre-defined study protocol [2]. The authors developed a self-administered e-survey on Google Forms (Google Inc., CA, USA) composed of 32 closed-ended, open-ended, and Likert Scale questions divided into four sections: sociodemographic characteristics (7 questions); definition of neurosurgical care (2 questions); knowledge of neurosurgical diseases, practice, and availability (11 questions); and common beliefs about neurosurgical care (12 questions). The surveys were in English and French S1 and S2 Files.

The study questions were adapted from a previous study on awareness, availability, and willingness [12]. Next, the questionnaire was validated by African stakeholders (neurosurgeons and patients) and piloted among 20 medical students. Cronbach’s alpha was used to evaluate the questionnaire’s internal consistency and principal component analysis to identify factor loadings. The survey was distributed daily via WhatsApp (WhatsApp, USA) to a sample of adult (>18 years) Africans from May 10, 2021, to July 14, 2021.

### Study population

The authors used the snowball sampling technique to recruit respondents. That is, the researchers identified WhatsApp groups of the target population and approached shared the links so willing respondents could opt-in the survey. Survey respondents were encouraged to share the link with their acquaintances within other WhatsApp groups and share the group size with the authors. The number of participants in each group was recorded to calculate the response rate. All consenting layperson adult Africans (i.e., not studying towards a healthcare degree or working in the healthcare sector) were deemed eligible. The authors chose to exclude medical/dental/nursing/allied health students and workers because this group will have better knowledge and more positive attitudes towards neurosurgical care than the general public—constituting a selection bias. The minimum sample size (N = 424) was determined using the normal approximation to the hypergeometric. Sample size calculations can be found in the study protocol [2].

### Outcome measures

The primary outcome was the level of knowledge about the availability of neurosurgical services. Secondary outcomes included level of knowledge in regards to neurosurgical disease in general as well as that seen in African neurosurgical practice.

### Statistical analysis

Independent variables included sociodemographic data and previous experience with neurosurgery. Dependent variables included the level of knowledge and willingness to use neurosurgical services. The definition of neurosurgery was considered correct when the free-text answer mentioned one or multiple nervous system components and surgery. Likert scale questions were coded as follows: strongly disagree = -2, disagree = -1, neutral = 0, agree = 1, and strongly agree = 2. SPSS version 26 (IBM, WA, USA) was used for statistical analysis which included univariable (i.e., frequencies and percentages for qualitative variables and means with their 95% confidence intervals [95% CI] for quantitative variables), bivariable (i.e., odds ratios and their 95% CI, Chi-Square test, and ANOVA), and multinomial regression analyses.

### Ethics

The e-survey was anonymous and voluntary. It therefore met the informed consent waiver requirements of the institutional review board of the Bel Campus University of Technology, Kinshasa, DRC (No 2135488) [2]. Study participants were informed of the aim and nature of the study. Informed consent was sought and respondents who did not consent were immediately redirected to the end of the survey. Participants were equally informed of their right to withdraw at any point of the study without risk of prejudice. The authors maintained confidentiality throughout the study and study data was only accessible on a need-to-know basis via a password-protected account.

## Results

The survey had a high-reliability score for neurosurgeon trust level (7 items, Cronbach’s alpha = 0.74). Eight hundred and three of the 1024 adult Africans approached responded to the survey, that is a 78.42% response rate. Six hundred and fifty-two of 803 responses (81.20%) were included in the final analysis. The reasons for exclusion were: respondents consented but were studying or working in a health facility (n = 141, 17.56%) and ten (1.24%) respondents did not give consent.

### Sociodemographic factors

Responses from 662 (82.4%) adult Africans aged 25.4 (95% CI: 25.0, 25.9) years were included in our analysis. Majority of respondents were male (n = 363, 54.8%), single (n = 574, 86.7%), urban dwellers (n = 600, 90.6%), English speakers (n = 506, 76.4%). Nigeria (n = 160, 24.2%), Rwanda (n = 112, 16.9%), the Democratic Republic of Congo (DRC) (n = 107, 16.2%), Cameroon (n = 105, 15.9%), and Zambia (n = 102, 15.4%) each had more than 100 responses (Table 1).

**Table 1:** Sociodemographic characteristics of survey respondents.

Characteristics	Frequency (Percentage)
**Sex**	
Female	299 (45.2)
Male	363 (54.8)
**Marital status**	
Single	574 (86.7)
Married, divorced, widowed	88 (13.3)
**Language**	
English	506 (76.4)
French	156 (23.6)
**Nationality**	
Nigeria	160 (24.2)
Rwanda	112 (16.9)
Democratic Republic of Congo	107 (16.2)
Cameroon	105 (15.9)
Zambia	102 (15.4)
Uganda	35 (5.3)
Kenya	16 (2.4)
Botswana	9 (1.4)
Côte d’Ivoire	5 (0.8)
Burundi	3 (0.5)
Ghana	3 (0.5)
Central African Republic	1 (0.2)
Ethiopia	1 (0.2)
Niger	1 (0.2)
Sao Tome and Principe	1 (0.2)
Tanzania	1 (0.2)
**Residence**	
Urban	600 (90.6)
Rural	62 (9.4)
**Neurosurgical experience**	
Personal neurosurgical experience	9 (1.4)
Family neurosurgical experience	143 (21.6)

### Definition of neurosurgery and knowledge level

**Fig 1:**
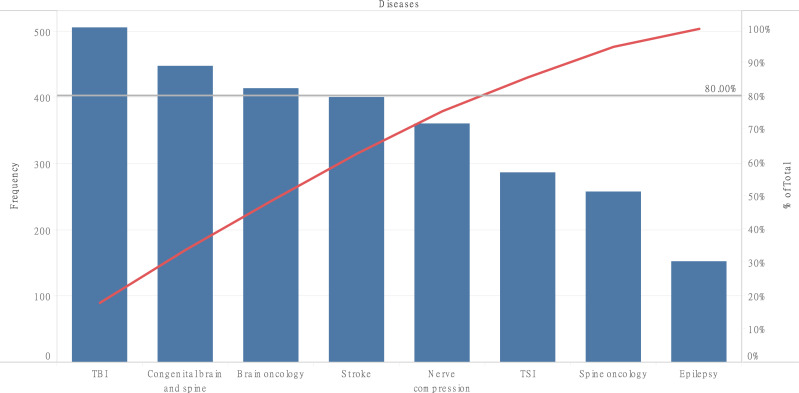
Pareto chart of diseases amenable to neurosurgery recognized by survey respondents. The black line shows the 80% cut-off while the red line shows the cumulative percentage total.

Most respondents (n = 505, 76.3%) gave a complete definition of neurosurgery (Table 2). Numerous definitions limited neurosurgery to brain surgery (n = 431, 65.1), while few definitions mentioned nerves (n = 50, 7.6%), and the spine (n = 20, 3.0%). Respondents recognized the following diseases treated by neurosurgeons: traumatic brain injury (TBI) (n = 505, 76.3%), congenital brain and spine diseases (n = 448, 67.7%), brain oncology (n = 414, 62.5%), and stroke (n = 400, 60.4%) (Fig 1). Forty-five (6.8%) respondents identified all the diseases amenable to neurosurgery while three (0.5%) failed to identify a single disease (Table 2). Urban dwellers recognized more neurosurgical diseases than rural dwellers (4.3 vs. 3.7, F = 5.11, P = 0.02). Similarly, patients with a neurosurgical family history identified more diseases (4.6 vs. 4.2, F = 5.12, P = 0.02).

**Table 2:** Knowledge levels of survey respondents.

Concept evaluated	Frequency (Percentage)
**Definition of neurosurgery**	
Complete	505 (76.3)
Incomplete	133 (20.1)
Incorrect	24 (3.6)
**Number of diseases amenable to neurosurgery correctly identified**	
8	45 (6.8)
7	87 (13.1)
6	92 (13.9)
5	83 (12.5)
4	104 (15.7)
3	93 (14.0)
2	38 (5.7)
1	117 (17.7)
0	3 (0.5)
**Mean number of correctly identified diseases by country (SD)**	
Côte d’Ivoire	5.8 (1.1)
Ethiopia	5.0
Cameroon	4.6 (2.1)
Rwanda	4.5 (2.2)
Zambia	4.5 (2.3)
Ghana	4.3 (3.1)
Botswana	4.2 (2.3)
Congo, The Democratic Republic	4.2 (2.2)
Uganda	4.1 (2.0)
Niger	4.0
Tanzania	4.0
Nigeria	4.0 (2.2)
Burundi	3.7 (3.8)
Central African Republic	3.0
Sao Tome and Principe	3.0
Kenya	2.6 (1.6)
**Misidentified kidney diseases as neurosurgical diseases**	29 (4.4)
**Misidentified prostate diseases as neurosurgical diseases**	29 (4.4)
**Awareness of the availability of neurosurgeons in their country**	452 (68.3)

Incomplete definitions regularly failed to mention the surgical nature of neurosurgery (n = 125, 18.9%). Respondents mistook neurosurgery with neurology (n = 16, 2.4%), psychiatry (n = 5, 0.8%), and general surgery (n = 3, 0.5%). Respondents incorrectly identified prostate (n = 12, 1.8%), kidney (n = 12, 1.8%), or both organ diseases (n = 17, 2.6%) as a focus of neurosurgery.

**Fig 2:**
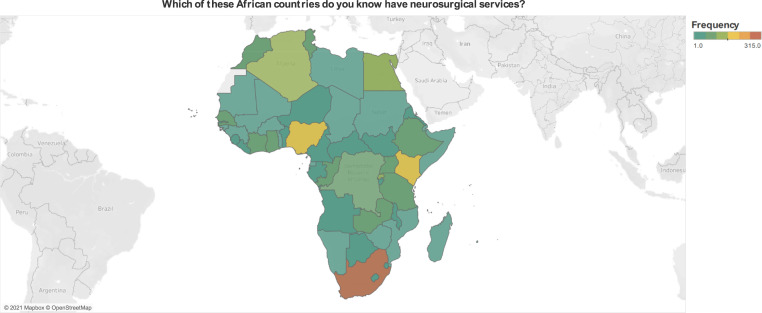
Respondents’ knowledge of neurosurgical care availability in Africa.

Sixty-six (10.0%) respondents did not know an African country offering specialized neurosurgical care. The most commonly cited African countries offering specialized care were: South Africa (n = 315, 47.6%), Nigeria (n = 172, 26.0%), Kenya (n = 158, 23.9%), Egypt (n = 143, 21.6%), Algeria (n = 131, 19.8%), Rwanda (n = 115, 17.4%), Ghana (n = 103, 15.6%), and Morocco (n = 92, 13.9%) (Fig 2).

### Attitude towards neurosurgery

**Fig 3:**
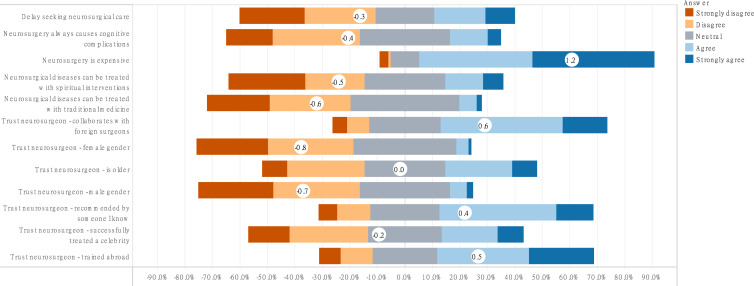
Diverging bar chart of Likert scale questions showing the percentage of respondents who agree or disagree with an assertion (bottom scale) and the average agreement score in white circles within the bar charts. Agreement levels were coded as Strongly disagree = -2, Disagree = -1, Neutral = 0, Agree = 1, and Strongly agree = 2. Hence, negative scores denote disagreement, and the more negative the score, the higher the disagreement. Similarly, positive scores represent agreement.

A handful of respondents believed it was important to consult a neurosurgeon as soon as possible (n = 194, 29.3%). Male respondents were more likely to consult a neurosurgeon early (F = 1.93, P = 0.04). Respondents trusted neurosurgeons more if they: collaborated with foreign neurosurgeons (n = 402, 60.7%), had trained abroad (n = 379, 57.2%), were recommended by the respondents’ acquaintance(s) (n = 371, 56.0%), were older (n = 221, 33.4%), or had treated a celebrity successfully (n = 196, 29.6%). The neurosurgeon’s gender did not influence the respondent’s trust in their provider’s skills (n = 247, 37.3%) (Fig 3). There were statistically significant gender differences in the respondents’ trust level towards their neurosurgeon.

We equally found statistically significant differences in trust level based on language differences. These included training abroad (F = 4.20, P<0.001), older age (4.16, P<0.001), recommendation by an acquaintance (F = 8.83, P<0.001), and female neurosurgeon gender (F = 0.02, P = 0.02). Being single was associated with higher trust levels towards neurosurgeons who have treated celebrities (F = 5.33, P = 0.01).

The majority of respondents believed neurosurgery is expensive (n = 566, 85.5%). This was especially true of respondents who had no personal neurosurgical history (F = 1.11, P = 0.003). These respondents were equally more likely to trust a neurosurgeon if they had trained abroad (F = 0.59, P = 0.006) or were older (F = 1.80, P = 0.02).

Respondents did not believe neurosurgical diseases could be treated with traditional medicine (n = 245, 52.1%) or by religious healers (n = 327, 49.4%). Most understood that cranial interventions did not necessarily lead to cognitive dysfunction (n = 322, 48.6%) (Fig 3).

### Willingness to use neurosurgical services

**Fig 4:**
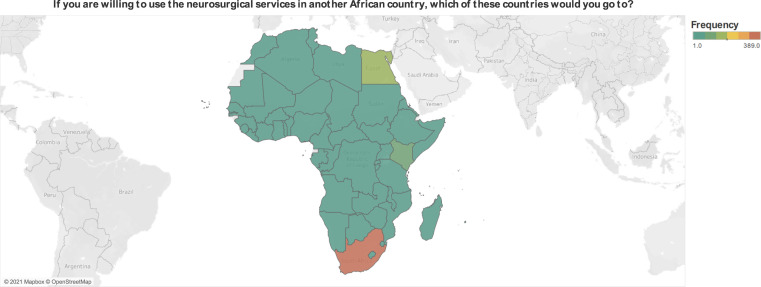
Respondents’ preferred destination for neurosurgical care.

Most respondents would use or recommend neurosurgical services in their country (n = 356, 53.8%), in other African countries (n = 505, 76.3%), and in non-African countries (n = 591, 89.3%). The most prevalent reason for not wanting to use African neurosurgical services was a lack of trust in the local infrastructure and skillset. Unwillingness to use or recommend in-country neurosurgical services was associated with the rural dwelling (β = -0.69, SE = 0.31, P = 0.03), lack of awareness about the availability of neurosurgeons (β = 1.02, SE = 0.20, P<0.001), and believing neurosurgery is expensive (β = -1.49, SE = 0.36, P<0.001). The most popular African destinations for neurosurgical care were: South Africa (n = 389, 58.8%), Egypt (n = 137, 20.7%), Kenya (n = 116, 17.5%), Nigeria (n = 65, 9.8%), Rwanda (n = 55, 8.3%), Ghana (n = 51, 7.7%), and Algeria (n = 50, 7.6%) (Fig 4). Unwillingness to use or recommend neurosurgical services in other African countries was associated with delays in seeking specialist care (β = 0.92, SE = 0.43, P = 0.03) and not believing all cranial operations result in cognitive deficits (β = -1.30, SE = 0.59, P = 0.03). Additionally, English-speaking participants (β = -1.15, SE = 0.34, P = 0.001), rural dwellers (β = 1.22, SE = 0.43, P = 0.01), and not delaying seeking specialist care (β = -1.44, SE = 0.61, P = 0.02) were associated with unwillingness to use or recommend neurosurgical services in non-African countries.

The most popular regions for medical tourism were Europe (n = 219, 33.1%), Asia (n = 135, 20.4%), North America (n = 127, 19.2%), Central America (n = 42, 6.3%), Australia (n = 18, 2.7%), and South America (n = 17, 2.6%). The majority of respondents reported they would prefer to seek neurosurgical care in Africa than outside Africa if barriers to care were eliminated (n = 618, 93.4%).

## Discussion

In this study, we evaluated the awareness, knowledge of availability, and willingness to use neurosurgical services on the African continent. Respondents had a good understanding of the scope of neurosurgery. However, the least recognized neurosurgical subspecialties were spine and functional surgery. Most respondents were aware of neurosurgical service delivery in their country and other African countries. However, a significant proportion of respondents were not willing to use neurosurgical services in their countries because they did not trust their neurosurgeons’ skill level or the local infrastructures. Respondents were more likely to trust their neurosurgeons if they regularly collaborated with foreign neurosurgeons, had trained abroad, and were recommended to the respondent by an acquaintance.

Samuel et al. found that most people prefer to consult a neurosurgeon when referred by their general practitioner, another healthcare professional, a family member, or a friend [14]. Other studies have found that the place of training and the geographic location of a neurosurgeon‘s practice are key determinants of the choice of neurosurgeon as patients prefer personnel who have trained abroad and practice in large urban academic hospitals [10, 14, 15]. In the past, few African countries had training programs so the majority of neurosurgeons trained abroad. However, Africa has experienced a growth in local training programs in the past decade [16]. As a result, fewer African neurosurgeons are training outside of Africa but more Africans are training in other African countries thanks to increased regionalized training such as with the College of Surgeons of East, Central, and Southern Africa and West African College of Surgeons and the availability of scholarships [16]. The preference for neurosurgeons trained abroad is due to a perception that training abroad is better. African neurosurgical educators can change this perception if they communicate on their training practices, operative accomplishments, academic output, and partnerships. Despite slight differences in the reasons advanced, it appears that physical appearance, race, and ethnicity play a minor role in the choice of a neurosurgeon among African adults [10]. Previous studies have found that non-African patients prefer older and professionally dressed neurosurgeons [10, 14, 15]. Of note, respondents reported that their neurosurgeon’s gender did not influence trust in their provider’s skills; however, male respondents were more likely to trust their neurosurgeon if they were male. These findings suggest unconscious gender bias among male patients who deny any bias when asked but have a higher likelihood to trust male providers.

Communication plays a crucial role in enabling populations to overcome biases and dispel ambiguity with regards to neurosurgical ailments and improving their knowledge of the scope, availability, and quality of care across their countries and the entire African continent. Although minimal, the confusion between neurosurgery and other specialties such as neurology and psychiatry highlights the need for further patient education. We suggest developing communication packages on neurosurgical diseases and scope of practice in local languages. Their content should be easy to understand, preferably with visual illustrations adapted to the lowest educational level [17]. These communication packages should be disseminated physically and online [18, 19]. It is critical that the information disseminated should not contain confusing medical terminology, extraneous or non-essential information, and information that might promote mistrust of health information or personnel [17, 20]. Neurosurgeons can learn lessons from the COVID-19 infodemic [20]. An Infodemic is characterized by false or misleading information in digital and physical environments during a disease outbreak causing confusion and risk-taking behaviors that can lead to mistrust of health authorities and undermine the public health response [20]. We have learned from the pandemic that communication efforts should be tailored to medically and informationally at-risk populations. For example, young adults might not be at-risk of a disease but they are at-risk of misinformation due to their social media usage. Hence, neurosurgeons must stress the importance of identifying fake news and the negative consequences of sharing them. Healthcare workers have a responsibility to listen to community concerns and questions; promote understanding of risks and advice from health experts; build resilience to misinformation; and engage and empower communities to take positive action [20–22].

Respondents perceived neurosurgery as being expensive and could constitute a reason for delayed care-seeking. Neurosurgeons should equally educate patients and communities on the costs of neurosurgical care. Particularly explaining concepts of out-of-pocket expenditure, financial risk protection, and cost-effectiveness. In addition, neurosurgeons should urge communities to take action by engaging policymakers to advocate for universal health coverage. It is essential that the data shared is contextually appropriate. Although this data exists for other surgical specialties and neurosurgical diseases in other regions, few studies have studied these concepts in Africa [23]. Hence, the African neurosurgical community should promote research such as cost-effectiveness analysis studies that focus initially on emergency and essentially neurosurgical interventions in Africa. This kind of data decreases misconceptions and guides health policy decisions and interventions [24–26].

Knowledge of the scope of neurosurgical procedures was limited to the brain. Notably, the least recognised sub-specialities were functional and spinal neurosurgery. These findings are noteworthy because both subspecialties address a significant proportion of disease burden. For example, we estimate that 1,032 spinal tumours, 70,032 traumatic spinal injuries, and 195,039 epilepsy cases require neurosurgical intervention in Africa [4]. Our findings suggest that some of the unmet spine and functional neurosurgery burden is unmet because African patients are not consulting neurosurgeons. Most respondents were aware of the availability of neurosurgeons or neurosurgery centers. This might be explained by the notable increase in the number of consulting neurosurgeons and training programs [18]. Despite this increase, many African countries including those of our respondents fall short of the recommended specialist neurosurgical workforce—Botswana (0.37), Cameroon (0.09), Ghana (0.07), Kenya (0.07), Rwanda (0.06), Côte d’Ivoire (0.05), Zambia (0.05), Niger (0.04), Ethiopia (0.03), Uganda (0.03), Burundi (0.02), Nigeria (0.02), Tanzania (0.02), DRC (0.01), and Sao Tomé et Principe (0.00) [19]. Of note, once we exclude our respondents’ countries from the list of most popular African countries offering neurosurgical care, the remaining countries (i.e., South Africa, Egypt, Algeria, and Morocco) are those with a storied reputation of quality neurosurgical care. The differences in trust levels between English-speaking and French-speaking (Cameroon, Côte d’Ivoire, Niger, Burundi, and DRC) respondents. These results suggest the need for culturally-appropriate communication.

We acknowledge a few limitations in this study. First, the majority of the data in this study originate from five countries only, hence the results may not be a true representation of African residents in general with most of the respondents being from urban zones. However, these countries represent the major African regions: Western (Nigeria), Eastern (Rwanda), Central (DRC and Cameroon), and Southern (Zambia) Africa. Second, additional variables such as health literacy level, educational level, socio-economic status, or history of a previous educational session on neurosurgery or on neurosurgical ailments; and variables as it relates to knowledge of availability and willingness to undergo treatment were not specifically explored and controlled for. We chose to exclude these variables to avoid having a long survey which would increase the risk of non-response, dropout, and survey fatigue [27]. An exploration of these additional associations may require future investigation.

## Conclusion

Respondents have a good understanding of the scope of neurosurgery and its availability in their countries but they are less likely to make use of these services. The lower willingness to use neurosurgical services can be attributed to a general lack of trust in local infrastructure. Also, most of the respondents believe neurosurgery is expensive. International collaboration, continued training, and proper modes of patient referral will improve trust levels and the use of neurosurgical services on the continent. Moving forward, research should focus on similar data obtained via means other than internet surveys to improve the accessibility to a wider audience, as well as cost-effectiveness in various aspects of neurosurgery.

## Supporting information

S1 File English survey. This file is the survey for this project in English.
(DOCX)

S2 File French survey. This file is the survey for this project in French.
(DOCX)
