# Supplementary material for: Public awareness, knowledge of availability, and willingness to use neurosurgical care services in Sub-Saharan Africa: A cross-sectional study
Source: PLoS One. 2022 Mar 17;17(3):e0264955. doi: 10.1371/journal.pone.0264955 (PMC8929639; doi:10.1371/journal.pone.0264955)
Supplement: S1 File — This file is the survey for this project in English. (DOCX) [file pone.0264955.s001.docx]

Public awareness, knowledge of availability, and readiness for neurosurgical care services in Africa

General Information

The aim of this study is to collect high quality and relevant data regarding the knowledge, availability, and readiness for neurosurgical services.

We appreciate your interest in participating in this questionnaire. You have been invited to participate as you are 18 years of age or over, and residing in Africa. Please read through this information before agreeing to participate by ticking the ‘yes’ box below.

You will be asked to answer questions about regarding the knowledge, availability, and readiness for neurosurgical services. This should take less than 10 minutes. No background knowledge is required. The data collected will be used for research purposes, data will be stored securely and accessible only by the primary researchers and users nominated by them. At the end of the project, the data will be stored for five years after final publication.

Do I have to take part?

Please note that your participation is voluntary. If you do decide to take part, you may withdraw at any point during the questionnaire for any reason before submitting your answers by pressing the closing the browser.

How will my data be used?

Your answers will be completely anonymous, and we will take all reasonable measures to ensure that they remain confidential.

Your data will be stored in a password-protected file and may be used in academic publications. Your IP address will not be stored. All questions are optional. Research data will be stored for a minimum of three years after publication or public release. The data that we collect from you may be transferred to, stored and/or processed at a destination outside your country and your continent. By submitting your personal data, you agree to this transfer, storing or processing.

Who will have access to my data?

Google is the data controller with respect to your personal data and, as such, will determine how your personal data is used. Please see their privacy notice here

h ttps://policies.google.com/privacy?hl=en-US. Google will share only fully anonymised data with all members of the research team for the purposes of this research.

We would also like your permission to use your anonymised data in future studies, and to share data with other researchers (e.g. in online databases). Any personal information that could identify you will be removed or changed before files are shared with other researchers or results are made public.

Who do I contact if I have a concern about the study or I wish to complain?

If you have a concern about any aspect of this study, please contact the principal investigator at [i kwueg buenyichibuikem@gmail.com](mailto:ikwuegbuenyichibuikem@gmail.com)

*Required

# Email address *

*Skip to question 2Skip to question 2*

By clicking the button below, you acknowledge that your participation in the study is voluntary, you are at least 18 years of age, and that you are aware that you may choose to terminate your participation in the study at any time and for any reason

Consent Form

1. Please indicate your consent before proceeding *

## Mark only one oval.

I consent *Skip to question 3*

I do not consent *Skip to section 8 (Completed)*

Eligibility criteria

1. Are you a health professional (physician, nurse, or allied health professional) or student in a health profession? *

## Mark only one oval.

Yes *Skip to section 8 (Completed)*

No *Skip to question 4*

Sociodemographic Characteristics

1. Age (Years) *

## Mark only one oval.

18

19

20

21

22

23

24

25

26

27

28

29

30

31

32

33

34

35

36

37

38

39

40

41+

# Sex *

## Mark only one oval.

Female Male

# Marital Status *

## Mark only one oval.

Married Single Separated Divorced Widowed

# Occupation/Profession *

1. Nationality *

## Mark only one oval.

Algeria Angola Benin Botswana Burkina Faso Burundi Cameroon Cabo Verde

Central African Republic Chad

Comoros

Congo, The Democratic Republic Congo, The Republic

Côte d’Ivoire Djibouti Equatorial Guinea Egypt

Eritrea Ethiopia Gabon Gambia Ghana Guinea

Guinea-Bissau Kenya

Lesotho, The Kingdom of Liberia

Libya

Madagascar Malawi

Mali Mauritania Mauritius Morocco Mozambique Namibia Niger Nigeria Rwanda

Sao Tome and Principe Senegal

Seychelles Sierra Leone Somalia South Africa South Sudan Sudan

Swaziland, Kingdom of Tanzania

Togo Tunisia Uganda Zambia Zimbabwe

# You live in an *

## Mark only one oval.

Urban area Rural area

# How long have you lived in the region ? *

## Mark only one oval.

Less than a year 1 year

- 1. years
  2. years
  3. years
  4. years
  5. years
  6. years
  7. years
  8. years
  9. years
  10. years
  11. years
  12. years
  13. years
  14. years
  15. years
  16. years
  17. years
  18. years
  19. years
  20. years
  21. years
  22. years
  23. years
  24. years
  25. years
  26. years
  27. years
  28. years
  29. years
  30. or more years

Definition

1. Please define neurosurgery or neurological surgery in your own words? *
2. Which of the following diseases can be treated by a neurosurgeon/neurological surgeon *

*Tick all that apply.*

Stroke or cerebrovascular accident

Cancers of the spine and spinal cord (ex: metastasis, ependymoma, meningioma) Traumatic brain/head injury (ex: epidural hematoma, subdural hematoma) Diseases of the kidney (ex: pyelonephritis, kidney cancer)

Traumatic spine injury (ex: spinal cord injury, epidural hematoma)

Diseases of the prostate (ex: benign prostate hyperplasia, prostatic cancer) Epilepsy (ex: epilepsy that cannot be treated with drugs)

Cancers of the brain (ex: meningioma, glioma, metastases) Compression of nerves (ex: sciatica)

Malformations of the brain and spine (ex: hydrocephalus, spina bifida)

Knowledge of neurosurgical disease, practice, and availability

"Neurosurgery or neurological surgery is the medical specialty concerned with the prevention, diagnosis, surgical treatment, and rehabilitation of disorders which affect any portion of the nervous system including the brain, spinal cord, central and peripheral nervous system, and cerebrovascular system." "Neurological Surgery Specialty Description". American Medical Association. Retrieved 4 October 2020.

1. Are you undergoing or have you undergone any neurosurgical treatment? *

## Mark only one oval.

Yes No

# Do you have or have you had any family members who has experiences of undergoing neurosurgical treatment *

## Mark only one oval.

Yes No

# Do you know or have you heard about neurosurgeons in your country? *

## Mark only one oval.

Yes No

# If there are neurosurgeons in your country do you know in which hospitals they work?

## Mark only one oval.

Yes No

# If you or someone you love needed neurosurgical care would you use the services in your country? *

## Mark only one oval.

Yes No

# Why? *

1. Which of these African countries do you know have neurosurgical services? *

*Tick all that apply.*

Algeria Angola Benin Botswana Burkina Faso Burundi Cameroon Cabo Verde

Central African Republic Chad

Comoros

Congo, The Democratic Republic Congo, The Republic

Côte d’Ivoire Djibouti Equatorial Guinea Egypt

Eritrea Ethiopia Gabon Gambia Ghana Guinea

Guinea-Bissau Kenya

Lesotho, The Kingdom of Liberia

Libya Madagascar Malawi

Mali Mauritania Mauritius Morocco Mozambique Namibia

Niger Nigeria Rwanda

Sao Tome and Principe Senegal

Seychelles Sierra Leone Somalia South Africa South Sudan Sudan

Swaziland, Kingdom of Tanzania

Togo Tunisia Uganda Zambia Zimbabwe

# If you or someone you love needed neurosurgical care would you use the services in another African country? *

## Mark only one oval.

Yes No

# Why? *

1. If you are willing to use the neurosurgical services n another African country, which of these countries would you go to?

*Tick all that apply.*

Algeria Angola Benin Botswana Burkina Faso Burundi Cameroon Cabo Verde

Central African Republic Chad

Comoros

Congo, The Democratic Republic Congo, The Republic

Côte d’Ivoire Djibouti Equatorial Guinea Egypt

Eritrea Ethiopia Gabon Gambia Ghana Guinea

Guinea-Bissau Kenya

Lesotho, The Kingdom of Liberia

Libya Madagascar Malawi

Mali Mauritania Mauritius Morocco Mozambique

Namibia Niger Nigeria Rwanda

Sao Tome and Principe Senegal

Seychelles Sierra Leone Somalia South Africa South Sudan Sudan

Swaziland, Kingdom of Tanzania

Togo Tunisia Uganda Zambia Zimbabwe

# Why?

1. If you or someone you love needed neurosurgical care would you use the services in a non-African country? *

## Mark only one oval.

Yes No

# Why? *

1. If you are willing to use the neurosurgical services of a non-African country, which region would you go to?

## Mark only one oval.

Australia Asia Europe

Central America North America South America

# Why?

1. If there was no obstacle which of the following neurosurgical services would you use first? *

## Mark only one oval.

In your country

In another African country In a non-African country

# Why? *

Common beliefs about neurosurgical care

1. You should only go to a neurosurgeon as a last resort or when surgery is needed *

## Mark only one oval.

Strongly disagree Disagree

Neutral Agree

Strongly agree

# Neurosurgery is expensive *

## Mark only one oval.

Strongly disagree Disagree

Neutral Agree

Strongly agree

# If my head is operated I will never be the same again *

## Mark only one oval.

Strongly disagree Disagree

Neutral Agree

Strongly agree

# I trust my neurosurgeon more if he/she has trained abroad *

## Mark only one oval.

Strongly disagree Disagree

Neutral Agree

Strongly agree

# I trust my neurosurgeon more if he/she is older *

## Mark only one oval.

Strongly disagree Disagree

Neutral Agree

Strongly agree

# I trust my neurosurgeon more if he/she has been recommended by someone I know *

## Mark only one oval.

Strongly disagree Disagree

Neutral Agree

Strongly agree

# I trust my neurosurgeon more if they he/she has successfully treated a celebrity (ex: politician, musician, wealthy individual) *

## Mark only one oval.

Strongly disagree Disagree

Neutral Agree

Strongly agree

# I trust my neurosurgeon more if she is male *

## Mark only one oval.

Strongly disagree Disagree

Neutral Agree

Strongly agree

# I trust my neurosurgeon more if she is female *

## Mark only one oval.

Strongly disagree Disagree

Neutral Agree

Strongly agree

# I trust my neurosurgeon more if he/she often collaborates with foreign surgeons *

## Mark only one oval.

Strongly disagree Disagree

Neutral Agree

Strongly agree

# 40. Neurosurgical diseases can be treated with traditional medicine *

## Mark only one oval.

Strongly disagree Disagree

Neutral Agree

Strongly agree

# 41. Neurosurgical diseases can be treated with spiritual intervention from religious leaders *

## Mark only one oval.

Strongly disagree Disagree

Neutral Agree

Strongly agree

Completed

Thank you for completing this survey
