# Supplementary material for: Public awareness, knowledge of availability, and willingness to use neurosurgical care services in Sub-Saharan Africa: A cross-sectional study
Source: PLoS One. 2022 Mar 17;17(3):e0264955. doi: 10.1371/journal.pone.0264955 (PMC8929639; doi:10.1371/journal.pone.0264955)
Supplement: S2 File — This file is the survey for this project in French. (DOCX) [file pone.0264955.s002.docx]

Sensibilisation du public, connaissance de la disponibilité et de la disponibilité des services de soins neurochirurgicaux en Afrique

Informations générales

L'objectif de cette étude est de recueillir des données pertinentes et de haute qualité concernant la connaissance, la disponibilité et la disponibilité des services de neurochirurgie.

Nous apprécions votre intérêt à participer à ce questionnaire. Vous avez été invité(e) à participer car vous êtes âgé(e) de 18 ans ou plus, et résidez en Afrique. Veuillez lire ces informations avant d'accepter de participer en cochant la case "oui" ci-dessous.

Il vous sera demandé de répondre à des questions concernant la connaissance, la disponibilité et la disponibilité des services de neurochirurgie. Cela devrait prendre moins de 10 minutes. Aucune connaissance de base n'est requise. Les données recueillies seront utilisées à des fins de recherche, elles seront stockées de manière sécurisée et ne seront accessibles qu'aux chercheurs principaux et aux utilisateurs qu'ils auront désignés. À la fin du projet, les données seront conservées pendant cinq ans après la publication finale.

Suis-je obligé de participer ?

Veuillez noter que votre participation est volontaire. Si vous décidez de participer, vous pouvez vous retirer à tout moment du questionnaire pour n'importe quelle raison avant de soumettre vos réponses en appuyant sur la touche de fermeture du navigateur.

Comment mes données seront-elles utilisées ?

Vos réponses seront totalement anonymes et nous prendrons toutes les mesures raisonnables pour en assurer la confidentialité.

Vos données seront stockées dans un fichier protégé par un mot de passe et pourront être utilisées dans des publications universitaires. Votre adresse IP ne sera pas conservée. Toutes les questions sont facultatives. Les données de recherche seront conservées pendant au moins trois ans après leur publication ou leur diffusion publique. Les données que nous recueillons auprès de vous peuvent être transférées, stockées et/ou traitées vers une destination située en dehors de votre pays et de votre continent. En soumettant vos données personnelles, vous acceptez ce transfert, ce stockage ou ce traitement.

Qui aura accès à mes données ?

Google est le responsable du traitement de vos données personnelles et, en tant que tel, il

déterminera l'utilisation de vos données personnelles. Veuillez consulter leur avis de confidentialité ici h ttps://policies.google.com/privacy?hl=en-US. Google ne partagera que des données entièrement anonymes avec tous les membres de l'équipe de recherche aux fins de cette étude.

Nous souhaitons également obtenir votre autorisation d'utiliser vos données anonymes dans le cadre d'études futures et de les partager avec d'autres chercheurs (par exemple, dans des bases de données en ligne). Toute information personnelle permettant de vous identifier sera supprimée ou modifiée avant que les fichiers ne soient partagés avec d'autres chercheurs ou que les résultats ne soient rendus publics.

Qui dois-je contacter si j'ai un problème avec l'étude ou si je souhaite déposer une plainte ?

Si vous avez un souci concernant un aspect quelconque de cette étude, veuillez contacter le chercheur principal à l'adresse [i kwueg buenyichibuikem@gmail.com](mailto:ikwuegbuenyichibuikem@gmail.com).

*Required

# Email address *

*Skip to question 2Skip to question 2*

En cliquant sur le bouton ci-dessous, vous reconnaissez que votre participation à l'étude est volontaire, que vous avez au moins 18 ans et que vous êtes conscient que vous pouvez choisir de mettre fin à votre participation à l'étude à tout moment et pour n'importe quelle raison.

Formulaire de consentement

1. Veuillez indiquer votre consentement avant de poursuivre *

## Mark only one oval.

Je consens *Skip to question 3*

Je ne consens pas *Skip to section 8 (Terminé)*

Critères d'éligibilité

1. Êtes-vous un professionnel de la santé (médecin, infirmier ou professionnel paramédical) ou un étudiant dans une profession de la santé ? *

## Mark only one oval.

Oui *Skip to section 8 (Terminé)*

Non *Skip to question 4*

Caractéristiques sociodémographiques

1. Âge (années) *

## Mark only one oval.

18

19

20

21

22

23

24

25

26

27

28

29

30

31

32

33

34

35

36

37

38

39

40

41+

# Sex *

## Mark only one oval.

Female Male

# État civil *

## Mark only one oval.

Marié(e) Célibataire Divorcé(e) Veuf(ve)

# Occupation/Profession *

1. Pays *

## Mark only one oval.

Algeria Angola Benin Botswana Burkina Faso Burundi Cameroon Cabo Verde

Central African Republic Chad

Comoros

Congo, The Democratic Republic Congo, The Republic

Côte d’Ivoire Djibouti Equatorial Guinea Egypt

Eritrea Ethiopia Gabon Gambia Ghana Guinea

Guinea-Bissau Kenya

Lesotho, The Kingdom of Liberia

Libya

Madagascar Malawi

Mali Mauritania Mauritius Morocco Mozambique Namibia Niger Nigeria Rwanda

Sao Tome and Principe Senegal

Seychelles Sierra Leone Somalia South Africa South Sudan Sudan

Swaziland, Kingdom of Tanzania

Togo Tunisia Uganda Zambia Zimbabwe

# Vous vivez dans une zone *

## Mark only one oval.

Urbaine Rurale

# Depuis combien de temps vivez-vous dans la région ? years=année) *

## Mark only one oval.

1

2

3

4

5

6

7

8

9

10

11

12

13

14

15

16

17

18

19

20

21

22

23

24

25

26

27

28

29

30

31 ou plus

Définition

1. Veuillez définir la neurochirurgie ou la chirurgie neurologique dans vos propres mots ? *
2. Parmi les maladies suivantes, lesquelles peuvent être traitées par un neurochirurgien/chirurgien neurologue ? *

*Tick all that apply.*

Accident vasculaire cérébral ou accident cérébrovasculaire

Cancers de la colonne vertébrale et de la moelle épinière (ex : métastase, épendymome, méningiome)

Lésion traumatique du cerveau/de la tête (ex : hématome épidural, hématome sous-dural) Maladies du rein (ex : pyélonéphrite, cancer du rein)

Lésion traumatique de la colonne vertébrale (ex : lésion de la moelle épinière, hématome épidural)

Maladies de la prostate (ex : hyperplasie bénigne de la prostate, cancer de la prostate) Épilepsie (ex : épilepsie qui ne peut être traitée par des médicaments)

Cancers du cerveau (ex : méningiome, gliome, métastases) Compression des nerfs (ex : sciatique)

Malformations du cerveau et de la colonne vertébrale (ex : hydrocéphalie, spina bifida)

Connaissance des maladies, de la pratique et de la disponibilité de la neurochirurgie.

"La neurochirurgie ou chirurgie neurologique est la spécialité médicale qui s'occupe de la prévention, du diagnostic, du traitement chirurgical et de la réadaptation des troubles qui affectent toute partie du système nerveux, notamment le cerveau, la moelle épinière, le système nerveux central et périphérique, et le système cérébro-vasculaire." "Description de la spécialité de la chirurgie neurologique". Association médicale américaine. Consulté le 4 octobre 2020.

1. Subissez-vous ou avez-vous subi un traitement neurochirurgical ? *

## Mark only one oval.

Oui Non

# Avez-vous ou avez-vous eu des membres de votre famille qui ont subi un traitement neurochirurgical ? *

## Mark only one oval.

Oui Non

# Connaissez-vous ou avez-vous entendu parler des neurochirurgiens dans votre pays ? *

## Mark only one oval.

Oui Non

# S'il existe des neurochirurgiens dans votre pays, savez-vous dans quels hôpitaux ils travaillent ?

## Mark only one oval.

Oui Non

# Si vous ou un de vos proches avait besoin de soins neurochirurgicaux, utiliseriez- vous les services de votre pays ? *

## Mark only one oval.

Oui Non

# Pourquoi ? *

1. Parmi ces pays africains, quels sont ceux qui, à votre connaissance, disposent de services de neurochirurgie ? *

*Tick all that apply.*

Algeria Angola Benin Botswana Burkina Faso Burundi Cameroon Cabo Verde

Central African Republic Chad

Comoros

Congo, The Democratic Republic Congo, The Republic

Côte d’Ivoire Djibouti Equatorial Guinea Egypt

Eritrea Ethiopia Gabon Gambia Ghana Guinea

Guinea-Bissau Kenya

Lesotho, The Kingdom of Liberia

Libya Madagascar Malawi

Mali Mauritania Mauritius Morocco Mozambique

Namibia Niger Nigeria Rwanda

Sao Tome and Principe Senegal

Seychelles Sierra Leone Somalia South Africa South Sudan Sudan

Swaziland, Kingdom of Tanzania

Togo Tunisia Uganda Zambia Zimbabwe

# Si vous ou l'un de vos proches aviez besoin de soins neurochirurgicaux, utiliseriez- vous les services d'un autre pays africain ? *

## Mark only one oval.

Oui Non

# Pourquoi ? *

1. Si vous êtes prêt à utiliser les services neurochirurgicaux d'un autre pays africain, dans lequel de ces pays iriez-vous ?

*Tick all that apply.*

Algeria Angola Benin Botswana Burkina Faso Burundi Cameroon Cabo Verde

Central African Republic Chad

Comoros

Congo, The Democratic Republic Congo, The Republic

Côte d’Ivoire Djibouti Equatorial Guinea Egypt

Eritrea Ethiopia Gabon Gambia Ghana Guinea

Guinea-Bissau Kenya

Lesotho, The Kingdom of Liberia

Libya Madagascar Malawi

Mali Mauritania Mauritius Morocco Mozambique

Namibia Niger Nigeria Rwanda

Sao Tome and Principe Senegal

Seychelles Sierra Leone Somalia South Africa South Sudan Sudan

Swaziland, Kingdom of Tanzania

Togo Tunisia Uganda Zambia Zimbabwe

# Pourquoi ?

1. Si vous ou l'un de vos proches aviez besoin de soins neurochirurgicaux, utiliseriez- vous ces services dans un pays non africain ? *

## Mark only one oval.

Oui Non

# Pourquoi ? *

1. Si vous êtes prêt à utiliser les services neurochirurgicaux d'un pays non africain, dans quelle région vous rendriez-vous ?

## Mark only one oval.

Australie Asie Europe

Amérique centrale Amérique du nord Amérique du sud

# Pourquoi ?

1. S'il n'y avait aucun obstacle, lequel des services neurochirurgicaux suivants utiliseriez-vous en premier ? *

## Mark only one oval.

Dans votre pays

Dans un autre pays d'Afrique Dans un pays non-africain

# Pourquoi ? *

Croyances communes sur les soins neurochirurgicaux

1. Vous ne devriez consulter un neurochirurgien qu'en dernier recours ou lorsqu'une intervention chirurgicale est nécessaire *

## Mark only one oval.

Pas du tout d'accord Pas d'accord

Neutre D'accord

Tout à fait d'accord

# La neurochirurgie est coûteuse *

## Mark only one oval.

Pas du tout d'accord Pas d'accord

Neutre D'accord

Tout à fait d'accord

# Si ma tête est opérée, je ne serai plus jamais le même. *

## Mark only one oval.

Pas du tout d'accord Pas d'accord

Neutre D'accord

Tout à fait d'accord

# Je fais davantage confiance à mon/ma neurochirurgien(ne) s'il/elle a été formé(e) à l'étranger. *

## Mark only one oval.

Pas du tout d'accord Pas d'accord

Neutre D'accord

Tout à fait d'accord

# Je fais davantage confiance à mon/ma neurochirurgien(ne) s'il/elle est plus âgé(e).

*

## Mark only one oval.

Pas du tout d'accord Pas d'accord

Neutre D'accord

Tout à fait d'accord

# Je fais davantage confiance à mon/ma neurochirurgien(ne) s'il/elle a été recommandé(e) par une personne que je connais. *

## Mark only one oval.

Pas du tout d'accord Pas d'accord

Neutre D'accord

Tout à fait d'accord

# Je fais davantage confiance à mon/ma neurochirurgien(ne) s'il/elle a traité avec succès une célébrité (ex : politicien, musicien, personne fortunée). *

## Mark only one oval.

Pas du tout d'accord Pas d'accord

Neutre D'accord

Tout à fait d'accord

# Je fais plus confiance à mon neurochirurgien s'il est un homme. *

## Mark only one oval.

Pas du tout d'accord Pas d'accord

Neutre D'accord

Tout à fait d'accord

# Je fais plus confiance à mon neurochirurgien s'il est une femme. *

## Mark only one oval.

Pas du tout d'accord Pas d'accord

Neutre D'accord

Tout à fait d'accord

# Je fais davantage confiance à mon/ma neurochirurgien(ne) s'il/elle collabore souvent avec des chirurgiens étrangers *

## Mark only one oval.

Pas du tout d'accord Pas d'accord

Neutre D'accord

Tout à fait d'accord

# Les maladies neurochirurgicales peuvent être traitées par la médecine traditionnelle *

## Mark only one oval.

Pas du tout d'accord Pas d'accord

Neutre D'accord

Tout à fait d'accord

# Les maladies neurochirurgicales peuvent être traitées grâce à l'intervention spirituelle des chefs religieux *

## Mark only one oval.

Pas du tout d'accord Pas d'accord

Neutre D'accord

Tout à fait d'accord

Terminé

Merci d'avoir répondu à cette enquête
